# Cape May as a Health Resort

**Published:** 1895-07

**Authors:** Albert E. Roussel

**Affiliations:** Assistant Professor of Practice and Clinical Medicine, Medico-Chirurgical College: Consulting Physician to the Temporary Home: Visiting Physician to the Howard Hospital


### Selection.

### CAPE MAY AS A HEALTH RESORT.

By ALBERT E. ROUSSEL, M. D.,

Assistant Professor of Practice and Clinical Medicine, Medico-Chirurgical College : Consulting Physician to the Temporary Home; Visiting Physician to the Howard Hospital.

As a summer resident of Cape May for some ten successive years, I have had a fairly good opportunity of judging of its merits as a health resort, more particularly during the summer months.

During this period of time I have been especially impressed by the marked improvement manifested in that very large group of cases that are presumably benefited by a seashore sojourn.

Curiously, however, there has undoubtedly existed a rather widespread impression that the relative humidity at this resort was higher than at some of its more populous rivals.

With the object of obtaining some definite data upon this subject I wrote for and procured the following interesting figures from the chief of the Weather Bureau at Washington.

As will be noticed from the figures, the relative annual percentage of humidity is but 77, as compared with 80 for Atlantic City, and no one monthly average proves an exception to this general rule.

A study of the temperature record is equally interesting. Although the mean annual temperature of Cape May is one and a fraction degrees higher than that of Atlantic City, yet it will be noted that the relative difference is but slightly marked during the summer and autumn months, but, on the contrary, is the most pronounced during the remaining portions of the year, which would only tend to emphasize the advantages of the Cape throughout the entire year.

This is especially true when we take into consideration the prevailing direction of the wind,—a point of no little importance during the summer season. On account of its insular position, the unwelcome land-breeze is a rare visitor, indeed, a direct northwest wind being alone responsible for its production.

Then, again, the manifest superiority of the magnificent beach, the absence of the particular crowds occasioned by cheap excursions, and last, but not least, the cleanly and well-kept streets, must certainly appeal to those who seek health as well as recreation.

UNITED STATES DEPARTMENT OF AGRICULTURE, }
WEATHER BUREAU, WASHINGTON, D. C., April 25, 1895. }

MARK W. HARRINGTON, Chief of Bureau.

*Mean Relative Humidity—Percentages.*—Atlantic City, N. J., January, 81 ; February, 79 ; March, 78 ; April, 77 ; May, 80 ; June, 82 ; July, 83 ; August, 83 ; September, 82 ; October, 80 ; November, 79 ; December, 80 ; annual, 80. Cape May, N. J., January, 78 ; February, 77 ; March, 76 ; April, 75 ; May, 77 ; June, 79 ; July, 80 ; August, 81 ; September, 77 ; October, 75 ; November, 73 ; December, 76 ; annual, 77.

*Monthly Mean Temperature.*—Atlantic City, N. J., January, 31.7 ; February, 33.1 ; March, 37.9 ; April, 46.5 ; May, 57 ; June, 66.7 ; July, 72.3 ; August, 72 ; September, 67.2 ; October, 57.1 ; November, 44.6 ; December, 35.6 ; annual, 51.8. Cape May, N. J., January, 34.2 ; February, 35.3 ; March, 39.7 ; April, 48.2 ; May, 58.7 ; June, 68.2 ; July, 73.6 ; August, 73.2 ; September, 68 ; October, 58.9 ; November, 46.4 ; December, 37.6 ; annual, 53.6.—*Medical Bulletin.*

---

SALIGENINE, according to Lederer (*Med. Bull.*), is of much value in treatment of acute articular rheumatism. It is almost free from drawbacks, and by its solubility in alkalies and its rapid absorption by the bowel may be utilized in other infectious diseases. It may be employed in relatively small does in comparison with salicylic acid. In proportion to the gravity of the case, it is prescribed in the dose of 50 centigrams to 1 gram (about 8 to 15 grains) every hour or two hours. Its action is rapid and stable. On account of the absence of buzzing in the ears, cyanosis, or digestive troubles, saligenine should, M. Lederer believes, be preferred to salicylic acid and its derivatives.—*Medical Standard.*

